# Gastric *Sarcina ventriculi*: A Report on Two Cases

**DOI:** 10.3390/reports8030128

**Published:** 2025-08-01

**Authors:** Yaomin Chen, Yu Liu, Zhiyan Fu

**Affiliations:** Department of Pathology, LSUHSC School of Medicine, New Orleans, LA 70112, USA; ychen3@lsuhsc.edu (Y.C.); yliu6@lsuhsc.edu (Y.L.)

**Keywords:** *Sarcina ventriculi*, gastric pathology, histological examination, antibiotics, case report

## Abstract

**Background and Clinical Significance**: *Sarcina ventriculi* is a rare Gram-positive coccus that thrives in acidic environments such as the human stomach. It has been increasingly identified in individuals with delayed gastric emptying and has been reported in association with various gastric disorders. However, its exact pathogenic role is not fully understood and remains controversial. **Case Presentation**: We present two cases of patients, one with a small bowel obstruction and the other with epigastric pain, both diagnosed with *Sarcina ventriculi* infection by histological examination of gastric biopsies. The patients were managed with a combination of antibiotics and a proton pump inhibitor, resulting in symptom resolution and clearance of *Sarcina ventriculi* upon follow-up examinations. **Conclusions**: This report explores the pathogenicity of *Sarcina ventriculi* by documenting its presence in symptomatic patients without other identifiable pathogens and demonstrating complete symptom resolution following targeted therapy. These findings raise the possibility of *Sarcina ventriculi*’s pathogenic potential under specific clinical conditions, suggesting it may act as more than a benign colonizer.

## 1. Introduction and Clinical Significance

*Sarcina ventriculi* is a rare, anaerobic, Gram-positive coccus predominantly found in soil and water, known for thriving in low-pH environments such as the human stomach [1,2,3]. It has been reported to be associated with various gastric disorders, such as emphysematous gastritis, gastric ulcers, and gastric perforation. Patients with underlying conditions that lead to gastric retention—such as diabetic gastroparesis, pyloric stenosis, gastric surgeries, or obstructive masses—are particularly susceptible to *Sarcina ventriculi* infection [4,5]. The gastric stasis in these conditions creates an ideal environment for bacterial overgrowth, facilitating the proliferation of *Sarcina ventriculi*. However, the exact role of *Sarcina ventriculi* in human disease remains uncertain. Some studies suggest it may act as a true pathogen responsible for serious gastric complications, while others consider it an incidental finding with unclear clinical significance [6,7]. In this report, we present two cases of *Sarcina ventriculi* infection to better understand its potential pathogenic role, as well as its clinical implications and management strategies.

## 2. Case Presentation

**Figure 1:**
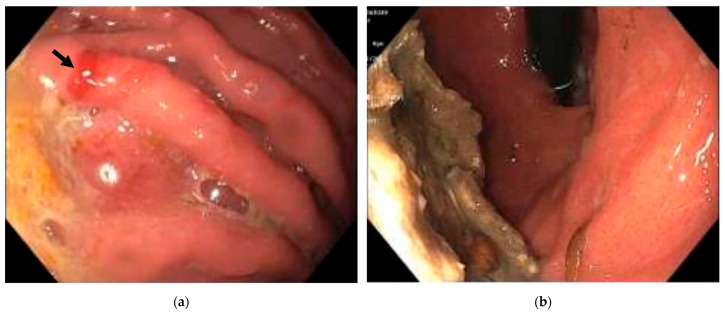
Esophagogastroduodenoscopy findings. (**a**) Gastric mucosal erosions with associated hemorrhage (arrow) in the gastric body, consistent with erosive gastropathy; (**b**) gastric mucosa with scattered inflammation and retained food, otherwise unremarkable following treatment.

**Figure 2:**
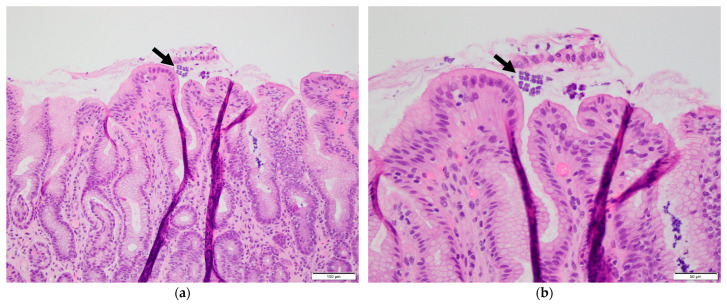
Gastric biopsy showing colonies of *Sarcina ventriculi* with characteristic tetrad arrangement (arrows). (**a**) Haematoxylin and eosin (H&E) stain, 200× magnification; (**b**) H&E stain, 400× magnification.

Case 1: A 69-year-old male with a history of small bowel obstruction and chronic inactive gastritis presented with abdominal pain and dark emesis lasting two days. The patient underwent esophagogastroduodenoscopy (EGD) within 24 h of admission to evaluate the hematemesis, which revealed several localized erosions with associated hemorrhages in the gastric body, consistent with erosive gastropathy (Figure 1a). Biopsies obtained for pathological evaluation revealed chronic focally active gastritis with basophilic tetrad microorganisms, morphologically consistent with *Sarcina ventriculi* (Figure 2). No *Helicobacter pylori* organisms were identified. The patient was promptly treated with a ten-day course of ciprofloxacin and proton pump inhibitor therapy with pantoprazole, and the symptoms were relieved. After two months, repeat EGD showed improved gastric mucosa with scattered inflammation but no evidence of erosive gastropathy (Figure 1b). Stomach biopsies were performed for re-examination, and *Sarcina ventriculi* was not detected.

Case 2: A 33-year-old male with a history of type 2 diabetes mellitus and epigastric burning presented with three days of epigastric pain and recurrent foul-smelling belching. An EGD was performed within 24 h of admission and revealed patchy mildly erythematous mucosa in the stomach. Gastric biopsies were obtained for histologic evaluation. Pathological examination showed minimal chronic inflammation and the presence of basophilic microorganisms forming characteristic tetrads, consistent with *Sarcina ventriculi*. No evidence of *Helicobacter pylori* was identified. The patient was promptly treated with a one-week course of ciprofloxacin, metronidazole, and pantoprazole. At the six-week follow-up, he reported resolution of symptoms. Repeat EGD revealed findings similar to the previous examination; however, biopsy showed no evidence of *Sarcina ventriculi*.

## 3. Discussion

*Sarcina ventriculi* is a Gram-positive, anaerobic coccus that thrives in acidic environments. It was initially recognized as a human pathogen in 1842 [8] and later successfully isolated from the stomachs of infected individuals in 1911 [9]. *Sarcina ventriculi*’s clinical relevance has remained a topic of debate since it can be found in both symptomatic and asymptomatic individuals. The bacterium thrives in conditions of reduced gastric acidity, such as hypochlorhydria or achlorhydria. While its presence in such cases may not always indicate active pathology and requires a thorough evaluation of the patient’s gastric environment and associated health conditions, *Sarcina ventriculi*’s ability to ferment carbohydrates produces gas, acid, and metabolic byproducts such as acetaldehyde and ethanol [10,11]. These substances can damage the gastric mucosa and induce injuries in the stomach and duodenum, thereby increasing the risk of severe complications including emphysematous gastritis and gastric perforation [12,13]. *Sarcina ventriculi* is also associated with delayed gastric emptying—including gastric outlet obstruction, gastroparesis, gastrointestinal surgeries, or gastric and pancreatic adenocarcinomas [14,15,16]—and case studies consistently show its frequent association with retained food during endoscopic evaluations, suggesting it may serve as a marker for impaired gastric motility [11,17].

In the two cases we presented, both patients had antecedent factors that may have altered gastric physiology and contributed to *Sarcina ventriculi* colonization. The first patient had a history of small bowel obstruction and chronic inactive gastritis, conditions known to impair gastric emptying and reduce acid production, potentially creating a favorable environment for *Sarcina ventriculi* proliferation. The second patient had type 2 diabetes mellitus and persistent epigastric symptoms, raising concern for diabetic gastroparesis, a recognized cause of delayed gastric transit and functional changes in acid secretion. Together, these cases suggest that impaired gastric motility and altered acid regulation may play a role in facilitating *Sarcina ventriculi* colonization and its possible pathogenic behavior.

Patients with *Sarcina ventriculi* colonization frequently present with nonspecific symptoms such as nausea, vomiting, dyspepsia, and abdominal discomfort [18]. However, reported symptoms vary widely, ranging from incidental findings in asymptomatic individuals to serious complications such as emphysematous gastritis and, in rare cases, life-threatening gastric perforation. Although its exact role in human disease is not fully understood, isolated cases of fatal gastric perforation attributed to *Sarcina ventriculi* indicate its potential to cause severe complications under certain conditions [19,20]. In contrast, our cases presented with mucosal injury without severe complications, and both patients responded well to medical therapy. These observations underscore the variable clinical spectrum of *Sarcina ventriculi*, which may range from asymptomatic colonization to significant pathology depending on the host context and underlying conditions.

While no laboratories are currently dedicated exclusively to *Sarcina ventriculi*, histopathological examination remains the primary diagnostic approach. Diagnosis is based on the bacterium’s distinctive tetrad morphology, characterized by large packets of basophilic spherical microorganisms approximately 3 μm in size, which is enhanced by hematoxylin and eosin, Gram, or Periodic acid–Schiff (PAS) staining [21,22]. However, identification can be challenging, as the organism’s small size and subtle appearance may lead to it being overlooked or misidentified, particularly with *Micrococcus* or *Staphylococcus* species [23]. Endoscopic findings associated with *Sarcina ventriculi* are variable, ranging from mild mucosal erythema to severe ulceration [24]. The two cases we reported included one with obvious gastric erosions and another with mildly inflamed gastric mucosa, both consistent with previously reported endoscopic findings. Definitive confirmation of the diagnosis can be made through polymerase chain reaction (PCR) or gene sequencing targeting the *Sarcina* 16S ribosomal RNA gene or pyruvate decarboxylase (PDC) gene [5,25,26], which allows for the precise identification of this bacterium, particularly in ambiguous cases. In our cases, the diagnosis was based solely on histological morphology due to limited access to molecular techniques, which may represent a potential diagnostic limitation.

Managing *Sarcina ventriculi* involves antibiotic therapy, particularly in cases associated with mucosal ulceration, to reduce the risk of serious complications such as gastric perforation, as well as addressing underlying gastric abnormalities to ensure effective treatment. Commonly used antibiotics include metronidazole, ciprofloxacin, piperacillin-tazobactam, ceftriaxone, and gentamicin, among others, with a median treatment duration of approximately 12 days (interquartile range: 7–28 days), as reported in the literature [23,27,28]. The choice and duration of antibiotic therapy may vary depending on clinical presentation and disease severity, as reflected in our two cases. Nevertheless, no consensus has been established regarding a standardized treatment regimen or duration. Surgical interventions may be necessary for anatomical obstructions [29], and prokinetic agents are often used to enhance gastric motility [30]. Proton pump inhibitors are frequently used to reduce acidity and relieve symptoms. However, recurrence is possible if underlying conditions persist, emphasizing the need for long-term management.

Although the pathogenicity of *Sarcina ventriculi* remains controversial, our clinical experience suggests a potentially causal role. In our cases, prompt antimicrobial therapy led to rapid symptom resolution, and follow-up EGD showed improved or similar gastric mucosa, with histological examination confirming clearance of the organism. These findings raise the possibility that, under certain conditions, *Sarcina ventriculi* may contribute to disease as more than an incidental colonizer, highlighting the importance of early recognition and treatment.

## 4. Conclusions

*Sarcina ventriculi*, though historically regarded as a non-pathogenic colonizer, is increasingly recognized as a potential opportunistic pathogen, particularly in patients with impaired gastric motility or delayed gastric emptying. The presented cases illustrate a possible association between *Sarcina ventriculi*, erosive gastropathy, and nonspecific gastrointestinal symptoms, with clinical improvement observed following antibiotic and proton pump inhibitor therapy. These observations suggest that *Sarcina ventriculi* may have a contributory role in gastric pathology under certain clinical conditions—raising the possibility of pathogenic potential in select scenarios. Clinicians may consider *Sarcina ventriculi* in differential diagnosis for patients presenting with gastric stasis, particularly when other common causes are excluded—even in the absence of specific symptoms. Future investigations should aim to clarify its pathogenic role in gastric diseases, establish standardized diagnostic criteria, and develop consensus treatment guidelines to optimize clinical outcomes.

## Data Availability

Data are contained within the article, and further inquiries can be directed to the corresponding author.
